# Supplementary material for: Time to revisit the skills and competencies required to work in rural general hospitals
Source: PLoS One. 2020 Oct 8;15(10):e0240211. doi: 10.1371/journal.pone.0240211 (PMC7544037; doi:10.1371/journal.pone.0240211)
Supplement: S1 Appendix — (DOCX) [file pone.0240211.s001.docx]

**S1 Appendix: Consultant and trainee Questionnaires**

**Consultant Questionnaire**

Demographics

1. Age
   1. 30-39
   2. 40-49
   3. 50-59
   4. 60-69
2. Gender
3. Where are you working currently?
4. Were you brought up in a remote and rural area?

Experience and Training

1. What specialist medical training did you undergo?
2. How long have you been a consultant in your main speciality?
3. How long have you worked in your current clinical post?
4. Do you have a planned age of retirement?
5. Have you previously worked in a remote and rural area?

Experience of current role

1. What attracted you to your current clinical role when you applied?
2. What do you think the positive aspects of this role are?
3. What do you think the negative aspects of this role are?
4. How would you describe your job to another medical consultant who is not working in a remote and rural setting?
5. What factors reduce any sense of professional isolation in your role?
6. What factors increase any sense of professional isolation in your role?

Remote and Rural Training pathway?

1. What training experience(s) were most useful to you before coming to this post?
2. Are there training experiences that you didn’t have, which you feel would have been useful before coming to work in a remote and rural environment?
3. Do you think there should be a Remote and Rural Medical training pathway
4. How would it differ from existing medical training pathways
5. Do you think this would affect recruitment to remote and rural medical posts
6. What do you think could be done to improve recruitment to remote and rural sites

**Junior Doctor Questionnaire**

**Demographics**

1. Age
   1. 20-29
   2. 30-39
   3. 40-49
   4. 50-59
   5. 60-65
2. Gender
3. Where are you currently working
4. Were you brought up in a remote and rural area?

**Experience and training**

1. What stage of training are you at?
2. Have you previously worked in a remote and rural area?
3. Did your medical school give the option of remote and rural placements, and did you undertake these?
4. Were you involved in any remote and rural student societies?

**Experience of current roles and future career plans**

1. What is your current role?
2. Did you specifically apply to your current job to work in remote and rural settings, or is it part of a larger training pathway?
3. Do you plan to work in remote and rural settings in the future?
   1. (If yes) When, where and why? And what factors are generally attractive about working in remote and rural settings
   2. (If no) Why? And what factors deter from working in remote and rural settings
4. What benefits do you think there are in working in a remote and rural setting?
5. What do you think could improve remote and rural working for trainees

**Remote and rural training pathway**

1. What training experience(s) were most useful to you before coming to this post?
2. Are there training experiences that you didn’t have, which you feel would have been useful before coming to work in a remote and rural environment?
3. Do you think there should be a Remote and Rural Medical training pathway?
4. How would it differ from existing medical training pathways?
5. Would you apply if it existed?
6. Do you think this would affect recruitment to remote and rural medical posts?
7. What do you think could be done to improve recruitment to remote and rural sites?
